# Aerobic capacity and muscle proteome: Insights from a mouse model

**DOI:** 10.1113/EP092308

**Published:** 2024-11-21

**Authors:** Abel Plaza‐Florido, Alejandro Santos‐Lozano, Susana López‐Ortiz, Beatriz G. Gálvez, Joaquín Arenas, Miguel A. Martín, Pedro L. Valenzuela, Tomàs Pinós, Alejandro Lucia, Carmen Fiuza‐Luces

**Affiliations:** ^1^ Pediatric Exercise and Genomics Research Center, Department of Pediatrics, School of Medicine University of California Irvine Irvine California USA; ^2^ i+HeALTH European University Miguel de Cervantes Valladolid Spain; ^3^ Department of Biochemistry and Molecular Biology, Faculty of Pharmacy Universidad Complutense de Madrid Madrid Spain; ^4^ Physical Activity and HEalth Reseach Group (PAHERG) Research Institute of the Hospital 12 de Octubre (‘imas12’) Madrid Spain; ^5^ Unit 701 Spanish Network for Biomedical Research in Rare Diseases (CIBERER) Madrid Spain; ^6^ Department of Systems Biology Universidad de Alcalá Madrid Spain; ^7^ Mitochondrial and Neuromuscular Disorders Unit, Vall d'Hebron Institut de Recerca Universitat Autònoma de Barcelona Barcelona Spain; ^8^ Faculty of Sport Sciences Universidad Europea de Madrid Madrid Spain; ^9^ Centre of Energy Environment and Technical Research (CIEMAT) Madrid Spain

**Keywords:** cardiorespiratory fitness, endurance, exercise, glycogenosis type V, omics, proteome

## Abstract

We explored the association between aerobic capacity (AC) and the skeletal muscle proteome of McArdle (*n* = 10) and wild‐type (*n* = 8) mice, as models of intrinsically ‘low’ and ‘normal’ AC, respectively. AC was determined as total distance achieved in treadmill running until exhaustion. The quadriceps muscle proteome was studied using liquid chromatography with tandem mass spectrometry, with the Search Tool for the Retrieval of Interacting Genes/Proteins database used to generate protein–protein interaction (PPI) networks and enrichment analyses. AC was significantly associated (*P*‐values ranging from 0.0002 to 0.049) with 73 (McArdle) and 61 (wild‐type) proteins (*r*‐values from −0.90 to 0.94). These proteins were connected in PPI networks that enriched biological processes involved in skeletal muscle structure/function in both groups (false discovery rate <0.05). In McArdle mice, the proteins associated with AC were involved in skeletal muscle fibre differentiation/development, lipid oxidation, mitochondrial function and calcium homeostasis, whereas in wild‐type animals AC‐associated proteins were related to cytoskeleton structure (intermediate filaments), cell cycle regulation and endocytic trafficking. Two proteins (WEE2, THYG) were associated with AC (negatively and positively, respectively) in both groups. Only 14 of the 132 proteins (∼11%) associated with AC in McArdle or wild‐type mice were also associated with those previously reported to be modified by aerobic training in these mice, providing preliminary evidence for a large divergence in the muscle proteome signature linked to aerobic training or AC, irrespective of AC (intrinsically low or normal) levels. Our findings might help to gain insight into the molecular mechanisms underlying AC at the muscle tissue level.

## Abstract (highlights)

- **What is the central question of this study?**
  What is the association between aerobic capacity (AC) and the skeletal muscle proteome of McArdle and wild‐type mice, as models of intrinsically ‘low’ and ‘normal’ AC, respectively?
- **What is the main finding and its importance?**
  In McArdle mice, the muscle proteins associated with AC were involved in skeletal muscle fibre differentiation/development, lipid oxidation, mitochondrial function and calcium handling. By contrast, in wild‐type mice AC‐associated proteins were related to cytoskeleton structure, cell cycle regulation and endocytic trafficking. On the other hand, there seems to be a divergence between the muscle proteome signature of AC and that previously reported to be linked to aerobic training. This work has provided preliminary evidence for the AC‐related muscle proteome signature.

## Graphical abstract

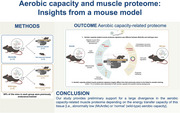

## 1 INTRODUCTION

There is a strong link between aerobic capacity (AC, the capacity to convert stored energy into movement (‘energy transfer’) through muscle oxidative phosphorylation (OXPHOS)) and overall health status (or the risk of morbidity/mortality) (Garton et al., 2016). Notably, the peak oxygen uptake (${\dot{V}}_{O_{2}\mathrm{peak}}$), also known as cardiorespiratory fitness (commonly abbreviated as CRF)—which is typically assessed during dynamic exercise until exhaustion and reflects an individual's peak capacity for energy transfer through OXPHOS—is a robust, independent prognostic factor of morbidity and mortality from all causes, particularly from cardiometabolic conditions (Fletcher et al., 2018; Lavie et al., 2019). Large‐scale clinical studies have in fact demonstrated that poor CRF is a better predictor of morbidity and mortality than some well‐established risk factors such as high blood pressure, type 2 diabetes, obesity or smoking (Kavanagh et al., 2003; Kokkinos et al., 2008; Myers et al., 2002), and the American Heart Association advocates for the routine assessment of this measure as a clinical vital sign (Ross et al., 2016). Furthermore, despite the strong evidence supporting the salutary effects of regular physical activity/exercise against the risk of major non‐communicable diseases, CRF per se (i.e., irrespective of physical activity levels) might represent an even more powerful health marker (Davidson et al., 2018; Myers et al., 2004).

Rodents represent an interesting model to resolve the complex link between AC and health/disease (Garton et al., 2016). Classic research by Koch and Britton (Koch et al., 2001) induced artificial selection of genetically heterogeneous rats over dozens of generations with the aim of developing strains that intrinsically differed in AC (as assessed during treadmill running until exhaustion). This approach showed that the risk of numerous non‐communicable conditions segregates with low and high AC (i.e., low and high resistance to disease, respectively) (Garton et al., 2016). Another approach is the use of genetically engineered mouse models. These animals can be generated and compared under identical genetic and environmental background to model human phenotypes and mechanisms; this homogeneous in vivo approach has proven resourceful for human research, including the use of rare or ‘extreme’ phenotypes (e.g., exceptionally low (or high) energy transfer capacity) (Garton et al., 2016). One such phenotype is McArdle disease (glycogen storage disease type V; OMIM database number 232600, ORPHA:368; Santalla et al., 2014).

McArdle disease (estimated prevalence of ∼1/140,000, with a similar sex distribution; Santalla et al., 2017) is an autosomal recessive disorder caused by pathogenic mutations in the gene (*Pygm*) encoding the skeletal muscle‐specific isoform of glycogen phosphorylase (myophosphorylase) (Villarreal‐Salazar et al., 2022), the enzyme that initiates glycogen breakdown in this tissue. Patients are therefore unable to obtain energy from this major substrate store and this condition represents the paradigm of ‘exercise intolerance’ (Santalla et al., 2014). Their ${\dot{V}}_{O_{2}\mathrm{peak}}$ is usually remarkably lower (–50–55%) than age and sex‐matched normative values, with only a very small proportion of patients (8%) showing values above the minimum threshold for cardiometabolic health, that is, 8 metabolic equivalents of task (commonly abbreviated as ‘MET’) (Villarreal‐Salazar et al., 2022). The finding of a low ${\dot{V}}_{O_{2}\mathrm{peak}}$ in patients with McArdle disease has been replicated in a genetically engineered (‘knock‐in’) mouse model (i.e., ∼50% lower AC than wild‐type values) (Brull et al., 2015). These mice, which harbour a common *Pygm* pathogenic genotype in patients (p.R50*/p.R50*), present indeed with a disease phenotype that closely mimics that of affected individuals, thereby representing an accurate study model of McArdle disease (Nogales‐Gadea et al., 2012).

High‐throughput, high‐sensibility proteome analysis (e.g., liquid chromatography with tandem mass spectrometry; LC‐MS/MS) allows identification of thousands of proteins in a given biological sample and is considered at the forefront of disease biomarker discovery (Larsen et al., 2023; Mann et al., 2021). This approach is used to study the biological mechanisms underlying exercise‐related phenotypes at different tissue levels (Sanford et al., 2020). For instance, a recent proteome study in rat skeletal muscle found AC to be positively associated with a higher abundance of proteins involved in β‐oxidation (Sadler et al., 2023). The main aim of the present study was to explore the association between AC and the skeletal muscle proteome in each of two different groups of mice, McArdle and wild‐type (i.e., as genetically homogeneous models of ‘very (unusually) low’ and ‘medium (normal)’ AC, respectively) using LC‐MS/MS. A secondary aim was to determine whether the AC‐linked muscle proteome was different from that previously shown to be induced by aerobic training in each these two mouse groups (Fiuza‐Luces et al., 2018). Our main hypothesis was that AC levels are overall associated with different muscle proteome signatures in the two groups owing to the limit in energy transfer at the skeletal muscle tissue level that is unique to McArdle disease.

## 2 METHODS

### 2.1 Ethical approval

This is an ancillary analysis of a previous study that received ethical institutional review board approval at that time (Centre of Energy, Environment and Technical Research (Madrid, Spain), reference number 179/15) (Fiuza‐Luces et al., 2018). All procedures were carried out according to European and Spanish legislative and regulatory guidelines (European convention ETS 123, on the use and protection of vertebrate mammals in experimentation and for other scientific purposes, and to the Spanish Laws 32/2007 and R.D. 1201/2005 on the protection and use of animals in scientific research). All possible efforts were made to minimize animal discomfort, and all the experiments were performed according to the animal ethical principles under which the journal operates.

### 2.2 Mice

A total of 18 C57Bl/6J male mice (10 McArdle and 8 wild‐type; all aged 16 weeks and with a body mass (mean ± SD) of 20.9 ± 1.0 and 22.1 ± 0.9 g, respectively, at the moment of sacrifice for muscle proteome determination) were included. Ten generations of backcrossing were performed onto the wild‐type C57Bl/6J background using the founder *Pygm* p.R50*/p.R50* knock‐in mice (Brull et al., 2015; Fiuza‐Luces et al., 2018; Nogales‐Gadea et al., 2012). All the animals were genotyped by PCR using the LoxP‐F and LoxP‐R primers as previously reported (Nogales‐Gadea et al., 2012). They were located in Eurostandard type IIL micro isolator cages (3–5 mice per cage) under controlled conditions of humidity and temperature (55 ± 10% and 20 ± 2°C, respectively). The mice maintained their circadian rhythm corresponding to light/darkness in periods of 12/12 h. The cages were lit (fluorescent lighting) from 07.00 h to 19.00 h and fluorescent lighting was also applied to treadmill sessions. All the mice were provided with ad libitum access to food (Teklad 2914; Harlan Teklad) and water (50 µm filtered and UV irradiated).

The proteome analyses (as described further below) of quadriceps muscle samples in the McArdle mice corroborated total deficiency of the *Pygm*‐encoded enzyme myophosphorylase (column CZA, Supplemental File 1), thereby ascertaining the presence of McArdle disease.

### 2.3 Aerobic capacity determination

All the mice had been previously familiarized with treadmill running (Harvard Apparatus; Panlab, Barcelona, Spain) as described elsewhere (Fiuza‐Luces et al., 2018). Thus, animals were allowed to adapt to the treadmill in three sessions (on three separate days). Adaptation involved a gradual increase in running time, treadmill velocity and inclination, starting with placement of the mouse on the treadmill with movement at a very low speed during the first day (0% inclination and 0–5 cm s^−1^ speed for 1 min, with 0.1 mA electrical stimulation) and ending with a 20‐min period at low running intensity on the third day (15% and 12 cm s^−1^, electrical stimulation 0.1 mA, 1 Hz, 200 ms). Four treadmills were used, and each mouse was consistently tested on the same treadmill.

All the mice performed two tests for AC determination, one at the age of 8 weeks (before the start of the 8‐week endurance training intervention, or the equivalent period in non‐trained mice, that is described below and after the aforementioned familiarization sessions) and one at the age of 16 weeks (after the 8‐week period). In the present study, only the results of the second AC test were used for studying the association between AC and the muscle proteome. The tests consisted of progressive exercise until exhaustion for determination of total running distance as a proxy for their AC (Høydal et al., 2007).

Following a warm‐up of 20 min (12 cm s^−1^, 15% inclination) the test for AC determination started at 5 cm s^−1^ (15% inclination) and the speed (but not the inclination) was gradually increased (3 cm s^−1^ every 2 min) until exhaustion (Fiuza‐Luces et al., 2018). The latter was determined when the mice spent more than 5 s continuously on the electric grid (electrical stimulation: 0.1 mA, 1 Hz, 200 ms) and were unable to continue running at the next speed (Ayala et al., 2009). All the tests were consistently performed between 08.00 and 12.00 h. We have used this test protocol for assessing AC in several previous studies with other types of mice debilitated by major conditions such as paediatric (Rincón‐Castanedo et al., 2023) or adult (Martín‐Ruiz et al., 2020) cancer, mitochondrial disorders (Fiuza‐Luces et al., 2019), or graft versus host disease (Fiuza‐Luces et al., 2013).

### 2.4 Previous aerobic training background

The aerobic training background was identical in the two study groups (McArdle and wild‐type mice), both in terms of the proportion of animals undergoing previous supervised aerobic training (i.e., 50% in each group) and in the characteristics (frequency, duration, relative intensity, load (volume and intensity) progression) of the programme in question—as reported in detail in our former paper (Fiuza‐Luces et al., 2018, Table A2). Thus, 50% of mice in each group had previously been trained during an 8‐week period (starting at the age of 8 weeks) at the same frequency (5 sessions/week, Monday to Friday, consistently performed between 08.00 and 12.00 h), volume (30 min per session at the start of the programme, then gradually increasing to 50 min by the end of it) and relative intensity—from 50% (start of the programme) to 70–75% of the maximal speed achieved (*V*_max_) at the end of the first AC test (end of the programme), with treadmill inclination gradually increasing from 0% (first day) to 15% (end of the programme). All the training sessions included a warm‐up period (15 min at 40% (start of the programme) to 50% of *V*_max_ (end)) and were followed by a cool‐down period (5 min at 35% of *V*_max_), both with the same treadmill inclination used for the core part. Of note, we have used a similar approach for increasing the relative intensity of aerobic (treadmill) training in previous studies in mice severely debilitated (and thus also with some degree of exercise intolerance) by other conditions such as paediatric cancer (Rincón‐Castanedo et al., 2023), mitochondrial disease (Fiuza‐Luces et al., 2019) or graft versus host disease (Fiuza‐Luces et al., 2013). In addition, all training sessions were performed in the same environmental (light, temperature, noise, humidity, handling) conditions. In order to minimize animal discomfort, only gentle tail touching was used to prompt the mice to run, and no electrical stimulation was applied during the training sessions. In addition, because carbohydrate ingestion 30–40 min prior to exercise attenuates the risk of muscle damage in McArdle disease (Lucia et al., 2021), for ethical reasons, during the hour before each session the McArdle mice were fed one Fruit Crunchies^TM^ pellet (weight, 190 mg; 52% energy from carbohydrate, 20.2% protein, 11.5% fibre, 6.3% fat, 5.1% ash, and <10% moisture) (Bio‐Serv^TM^, LBS (Serving Biotechnology), UK, London). We verified that all exercise sessions started after the mice had eaten the pellet (which consistently took ≤60 min). Ingestion of pellets was chosen instead of parenteral administration of glucose to minimize animal discomfort. To ensure similar conditions in the two groups, all the study mice (trained or not) consumed the aforementioned pellet at the same time of the day.

The rest of the mice (i.e., 50% in each group) did not perform treadmill exercise during the equivalent 8‐week period.

### 2.5 Quadriceps muscle tissue proteome

Two days after the second AC test, mice were killed (intraperitoneal injection of a lethal dose of Avertin (0.2%, 150 mg kg^−1^) followed by cervical dislocation to confirm animals’ death before tissue removal). The quadriceps muscles were then dissected, trimmed of connective tissue, and immediately snap‐frozen in liquid nitrogen before storage at −80°C for proteome analysis. A description of the protocol for studying the muscle proteome as previously reported by us (Fiuza‐Luces et al., 2018) is presented below.

Muscle tissue samples (∼140 mg) were extracted in lysis buffer (2% SDS, 10 mM Tris (2‐carboxyethyl) phosphine hydrochloride (TCEP) and 50 mM Tris–HCl, pH 7.5) by homogenizing the tissue with 3 cycles at 6500 rpm for 1 min, each using a MagNA Lyser Instrument (Roche; Mannheim, Germany). The samples were then boiled for 5 min and incubated for 30 min at room temperature with agitation. Thereafter, the samples were centrifuged at 16,000 *g* for 15 min, and the protein concentration in the supernatant was measured using a Direct Detect infrared spectrometer (Millipore Ibérica; Madrid, Spain).

For the quantitative differential analysis by LC‐MS/MS with isobaric tags (tandem mass tag (TMT) 10‐plex), ∼100 µg of total proteins was digested following a filter‐aided protocol, with slight modifications (Cardona et al., 2015). According to manufacturer's instructions, TM proteins were diluted in 7 M urea and 0.1 mM Tris–HCl (pH 8.5) and loaded onto 10 kDa centrifugal filter devices (NanoSep 10k Omega, Pall Life Sciences; Port Washington, NY, USA). The buffer was exchanged by washing the filters, and the proteins were alkylated using 50 mM iodoacetamide for 30 min in the dark. The excess of alkylating reagent was removed by washing three times with the buffer and three additional times with 50 mM ammonium bicarbonate. Proteins were then digested overnight at 37°C using modified trypsin (Promega Biotech Ibérica; Alcobendas, Madrid, Spain) in 50 mM ammonium bicarbonate at 30:1 protein:trypsin (w/w) ratio. The resulting peptides were eluted by centrifugation with 50 mM ammonium bicarbonate (twice) and 0.5 M sodium chloride. Trifluoroacetic acid (TFA) was added to a final concentration of 1% and the peptides were desalted using C18 Oasis‐HLB cartridges (Waters; Milford, MA, USA) and dried for further analysis.

For stable isobaric labelling, the resulting tryptic peptides were dissolved in 100 mM tri‐ethyl‐ammonium bicarbonate buffer, and the peptide concentration was determined by measuring amide bonds with the Direct Detect system (Millipore Ibérica). Equal amounts of each peptide sample were labelled using 10‐plex TMT Reagents (Thermo Fisher Scientific; Waltham, MA, USA) following the manufacturer's protocol. The peptides were labelled with TMT reagents that had been reconstituted in 70 µL of acetonitrile. According to the manufacturer (Thermo Fisher Scientific), the efficiency of TMT labelling of both N‐terminal and lysine residues in proteins is ≥99.5%—that is, almost all peptides will contain at least one tag, ensuring reliable quantification in LC‐MS/MS analyses. After a 2‐h incubation at room temperature, the reaction was halted by adding 0.5% TFA, followed by a 30‐min incubation, after which the peptides were combined. The samples were then concentrated in a Speed Vac, desalted using C18 Oasis‐HLB cartridges and dried for further analysis. To enhance proteome coverage, the TMT‐labelled samples were fractionated using high‐pH reverse‐phase chromatography (High pH Reversed‐Phase Peptide Fractionation Kit, Thermo Fisher Scientific) and concentrated as before.

The labelled peptides were analysed by LC‐MS/MS using a C‐18 reversed phase nano‐column (75 µm I.D. × 50 cm, 2 µm particle size, Acclaim PepMap RSLC 100 C18; Thermo Fisher Scientific) under a continuous acetonitrile gradient consisting of 0–30% B over 360 min and 50–90% B in 3 min (A is 0.1% formic acid; B is 90% acetonitrile, 0.1% formic acid). Peptides were eluted from the nano‐column at a flow rate of 200 nL min^−1^ into a nanospray emitter for real time ionization and fragmentation on an Orbitrap Fusion mass spectrometer (Thermo Fisher Scientific). The analysis included an enhanced Fourier transform‐resolution spectrum (resolution = 70,000) followed by MS/MS spectra of the *N*th most intense parent ions throughout the chromatographic run. Dynamic exclusion was set at 40 s.

For peptide identification, all spectra were analysed with Proteome Discoverer (version 2.1.0.81, Thermo Fisher Scientific) using the SEQUEST‐HT search engine (Thermo Fisher Scientific). Database searches were conducted against the Uniprot database, including all mouse sequences and contaminants (48,644 entries). The search parameters were set as follows: trypsin digestion allowing up to two missed cleavage sites, precursor and fragment mass tolerances of 2 and 0.02 Da, respectively, carbamidomethyl cysteine and TMT labelling at N‐terminal and Lys residues as fixed modifications, and methionine oxidation as a dynamic modification. Peptide identification was performed using the probability ratio method (Martínez‐Bartolomé et al., 2008) and the false discovery rate (FDR) was calculated using inverted databases and the refined method (Navarro & Vazquez, 2009) with additional filtering based on a precursor mass tolerance of 15 ppm (Bonzon‐Kulichenko et al., 2015).

Identified peptides with an FDR ≤ 1% were used to quantify the relative abundance of each protein from reporter ion intensities. Analysis of quantitative data was performed using the weighted spectrum, peptide and protein (‘WSPP’) model previously described (Navarro et al., 2014). In this model, protein log2‐ratios are expressed as standardized variables—that is, in units of standard deviation (SD) according to their estimated variances (*Z*_q_ values).

### 2.6 Statistical analyses

Between‐group (McArdle vs. wild‐type mice) differences in AC at the age 16 weeks (corresponding to the time of muscle proteome analyses) were assessed with the Mann–Whitney's *U*‐test.

Pearson correlations between AC and proteome data were performed separately for each mouse group using R (version 4.2.3; R Foundation for Statistical Computing, Vienna, Austria). Proteins significantly associated with AC (*P *< 0.05) were used to create two protein–protein interactions (PPI) networks (i.e., one for McArdle mice and one for wild‐type mice) using the Search Tool for the Retrieval of Interacting Genes/Proteins (STRING) database (http://string‐db.org/) (Szklarczyk et al., 2023). PPI networks help to understand the cellular and molecular processes at the systems level, suggesting novel directions for future experimental studies (Szklarczyk et al., 2023). In STRING settings, we selected a minimum interaction score of 0.150 and PPI enrichment *P *< 0.05. Enrichment analyses were performed in STRING to identify biological processes/pathways associated with AC levels in McArdle and wild‐type mice, respectively (FDR <0.05). A node degree (i.e., the number of interactions for each protein within the PPI) ≥10 was considered to highlight the hub proteins (i.e., the proteins located in the centre of the PPI networks).

We also performed *in silic*o data mining to elucidate relevant proteins associated with AC in the context of cardiometabolic disease and endurance training. This was conducted in three steps. First, we checked the overlap between proteins previously associated with McArdle disease in human skeletal muscle tissue (García‐Consuegra et al., 2022) and the list of skeletal muscle proteins that were associated with AC in our two mouse groups. Thereafter, we overlapped the proteins associated with AC in the two groups with 140 protein‐coding genes associated with type I and II diabetes using the PHENOPEDIA database (Yu et al., 2010). Lastly, we explored if aerobic training can alter the expression of the protein‐coding genes that were (i) associated with AC (in McArdle and wild‐type mice) in the present study and also (ii) altered by endurance training in McArdle and wild‐type mice in our previous report using the same proteome data (Fiuza‐Luces et al., 2018). For this purpose, we used an up‐to‐date database (https://extrameta.org/) derived from an extensive meta‐analysis of 43 publicly available transcriptome results from human skeletal muscle and blood in response to aerobic exercise interventions (Amar et al., 2021).

## 3 RESULTS

### 3.1 Aerobic capacity of McArdle and wild‐type mice

The aerobic capacity of McArdle mice at age 16 weeks was notably lower (–47%) compared to wild‐type mice (153 ± 54 and 291 ± 122 m, respectively; *P* = 0.010).

As reported elsewhere (Fiuza‐Luces et al., 2018), the previous 8‐week aerobic training programme performed by 50% of the mice in each group had induced significant improvements in the AC of both McArdle (+18%, *P* = 0.035) and wild‐type (+28%, *P* = 0.041) mice compared to age 8 weeks, versus no significant change during the same time period in the remainder (non‐trained) 50% of mice in each group (–17%, *P* = 0.129, and –2%, *P* = 0.726, respectively). The relative training‐induced improvement in AC did not significantly differ between McArdle and wild‐type mice (*P* = 0.111). Of note, the AC of the McArdle mice was notably lower vs. wild‐type mice, even when comparing the McArdle mice that had undergone the previous training programme with the non‐trained wild‐type mice (i.e., – 44%) (Fiuza‐Luces et al., 2018). Thus, despite a significant improvement in AC, aerobic training was clearly unable to rescue a ‘normal’ AC phenotype in McArdle mice, as also reported in patients (Santalla et al., 2022). McArdle disease therefore represents a paradigm of low AC irrespective of training status.

### 3.2 Association of muscle proteome with aerobic capacity: McArdle mice

**FIGURE 1:**
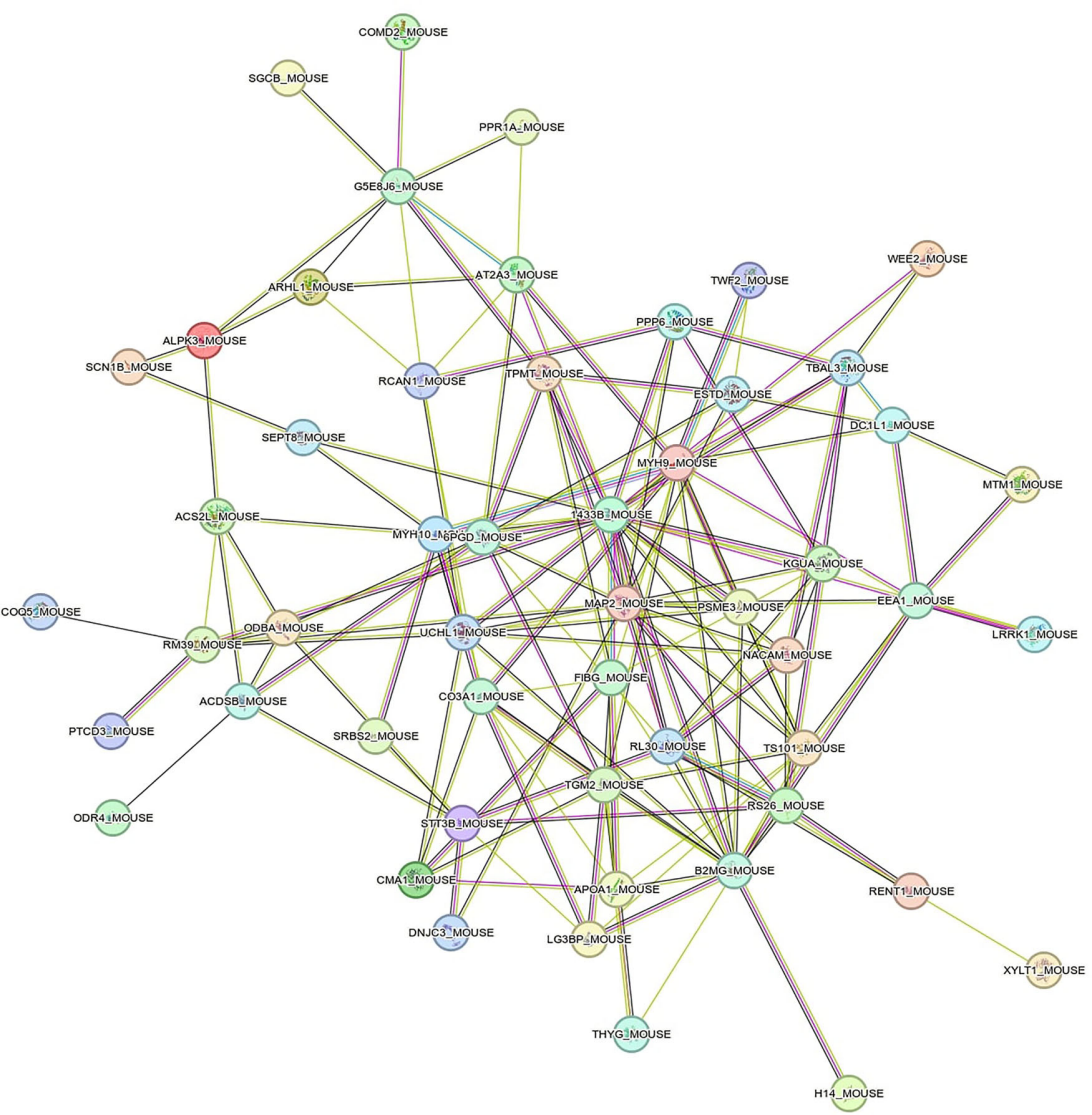
PPI network associated with aerobic capacity in McArdle mice. Each node represents a protein and the edges colour the type of relationship between each protein (i.e., node in the network). Purple line, experimental evidence; yellow line, textmining evidence; light blue line, database evidence; black line, coexpression evidence. No meaning is given to the node colour, which was assigned by default in the STRING database. PPI, protein–protein interaction; STRING, Search Tool for the Retrieval of Interacting Genes/Proteins.

**FIGURE 2:**
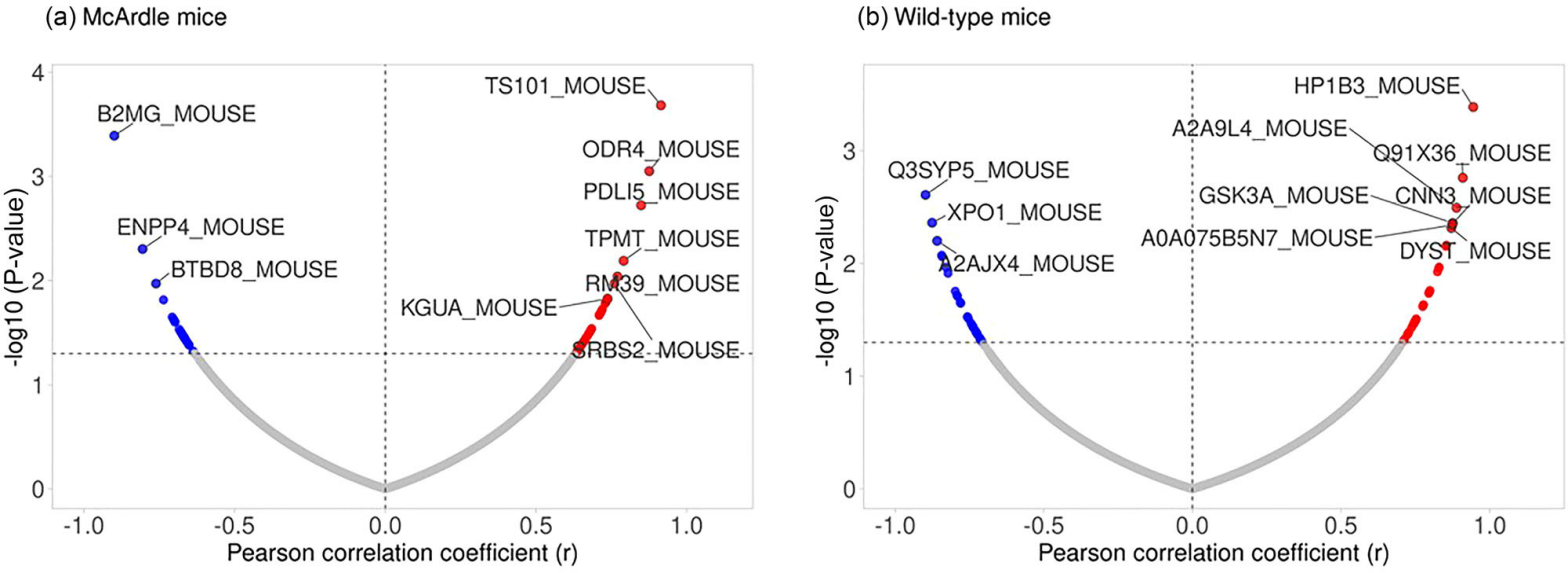
Volcano plots show the association between aerobic capacity and muscle proteome in McArdle (a) and wild‐type (b) mice. Statistically significant threshold is indicated in the *y*‐axis (i.e., *P*‐values ranging from 0.0002 to 0.049 correspond to –log10 between 1.301 and 3.683, in the horizontal dashed line). Protein names are presented for the 10 proteins (each point corresponds to one protein), with the higher correlation coefficient on the *x*‐axis (positive or negative values in red and blue points, respectively) in McArdle and wild‐type.

A total of 3237 proteins were detected in the skeletal muscle samples from McArdle mice. AC was significantly associated with 73 proteins in skeletal muscle (46 positive (*r* ranging from 0.63 to 0.91) and 27 negative (– 0.63 to – 0.89) associations, with individual *P*‐values ranging from 0.0002 to 0.049) (Figures 1 and 2a and Supplementary File 2). When further gene ontology (GO) analysis was performed using the STRING database (http://string‐db.org/) (Szklarczyk et al., 2023) as well as manual curation of the literature, it was observed that these proteins are mainly involved in the following:

Skeletal muscle fibre differentiation and skeletal muscle structure and development: myosin 9 (MYH9) and 10 (MYH10); ubiquitin carboxyl‐terminal hydrolase isozyme L1 (UCHL1); ADP‐ribosylarginine hydrolase‐like protein 1 (ARHL1); calcipressin‐1 (RCAN1); beta‐sarcoglycan (SGCB); α‐protein kinase 3 (ALPK3); sorbin and SH3 domain‐containing protein 2 (SRBS2); collagen α‐1(III) chain (CO3A1); and myotubularin 1 (MTM1).

Lipid oxidation and mitochondrial function: acetyl‐coenzyme A synthase 2‐like, mitochondrial (ACS2L); short/branched chain specific acyl‐CoA dehydrogenase, mitochondrial (ACDSB); protein‐glutamine γ‐glutamyltransferase 2 (TGM2); truncated apolipoprotein A‐I (APOA1); 2‐oxoisovalerate dehydrogenase subunit α, mitochondrial (ODBA); pentatricopeptide repeat domain‐containing protein 3 pentatricopeptide repeat domain 3 (PTCD3); and myotubularin 1 (MTM1).

Intracellular calcium handling: sarcoplasmic/endoplasmic reticulum calcium ATPase 3 (AT2A3); and histidine‐rich calcium‐binding protein (G5E8J6).

Intracellular signal transduction: protein phosphatase 1 regulatory subunit 1A (PPR1A).

Figure 1 shows the PPI network (number of nodes = 60; number of edges = 152; expected number of edges = 125; PPI enrichment—*P*‐value = 0.010). These proteins were enriched in GO biological processes such as muscle cell differentiation and structure, protein metabolic processes, as well as in compartments such as sarcoplasm and UniProt terms acetylation and phosphoprotein (FDR values ranging from 0.0003 to 0.044) (Table 1). The node degree ranged from 0 to 20 (mean = 5). Six proteins (MYH9; methionine aminopeptidase 2 microtubule associated protein 2 (MAP2); β‐2‐microglobulin (B2MG); 6‐phosphogluconate dehydrogenase, decarboxylating (6PGD); protein‐glutamine γ‐glutamyltransferase 2 (TGM2); and 4‐3‐3 protein beta/alpha, N‐terminally processed (1433B)) showed a node degree ≥10 (i.e., ≥10 interactions with other proteins in the PPI network) (Supplementary File 2).

**TABLE 1:** Functional enrichment analyses of proteins associated with aerobic capacity in McArdle mice using Search Tool for the Retrieval of Interacting Genes/Proteins (STRING) database (Szklarczyk et al., 2023).

Category	Selected terms	Number of proteins in each term	Matching proteins in the PPI network
GO process	Striated muscle cell differentiation	7	**MYH9**, **ARHL1**, RCAN1, SGCB, **MYH10**, ALPK3, SRBS2
GO process	Muscle structure development	10	**MYH9**, **UCHL1**, **ARHL1**, RCAN1, SGCB, CO3A1, **MYH10**, ALPK3, MTM1, SRBS2
GO process	Organonitrogen compound metabolic process	31	TS101, LRRK1, ACDSB, **MYH9**, PSME3, DNJC3, **CMA1**, **ENPP4**, RS26, PPP6, ACS2L, **UCHL1**, **XYLT1**, **ARHL1**, **APOA1**, STT3B, **WEE2**, MAP2, **H14**, THYG, ODBA, PTCD3, CO3A1, **TGM2**, **B2MG**, ALPK3, RL30, RM39, MTM1, KGUA, **FIBG**
GO process	Muscle tissue development	8	**ARHL1**, RCAN1, SGCB, CO3A1, **MYH10**, ALPK3, MTM1, SRBS2
GO process	Protein metabolic process	25	TS101, LRRK1, **MYH9**, PSME3, DNJC3, **CMA1**, RS26, PPP6, **UCHL1**, **XYLT1**, **ARHL1**, **APOA1**, STT3B, **WEE2**, MAP2, **H14**, PTCD3, CO3A1, **TGM2**, **B2MG**, ALPK3, RL30, RM39, MTM1, **FIBG**
GO component	Cytoplasm	50	TS101, LRRK1, ACDSB, **MYH9**, **1433B**, PSME3, AT2A3, **TBAL3**, TPMT, DNJC3, **CMA1**, PPR1A, TWF2, RS26, PPP6, ACS2L, **UCHL1**, **XYLT1**, **ARHL1**, **APOA1**, STT3B, DC1L1, **WEE2**, HTR5A, COQ5, MAP2, RCAN1, EEA1, THYG, ODBA, RENT1, SGCB, PTCD3, **6PGD**, **G5E8J6**, NACAM, **TGM2**, **B2MG**, **MYH10**, T10B, RL30, RM39, **SEPT8**, CA043, **COMD2**, MTM1, KGUA, SRBS2, ESTD, **FIBG**
GO component	Cytosol	27	TS101, LRRK1, **MYH9**, **1433B**, PSME3, DNJC3, **CMA1**, TWF2, RS26, PPP6, **UCHL1**, **APOA1**, **WEE2**, HTR5A, MAP2, EEA1, RENT1, PTCD3, **6PGD**, **TGM2**, **B2MG**, **MYH10**, RL30, CA043, MTM1, KGUA, ESTD
Reactome	Metabolism of proteins	15	PSME3, **TBAL3**, DNJC3, **CMA1**, RS26, PPP6, **UCHL1**, **APOA1**, DC1L1, PTCD3, **G5E8J6**, RL30, RM39, **COMD2**, **FIBG**
UniProt keywords	Acetylation	24	TS101, ACDSB, **MYH9**, **1433B**, PSME3, AT2A3, TPMT, PPR1A, TWF2, PPP6, ACS2L, STT3B, MAP2, **H14**, ODBA, PTCD3, **6PGD**, NACAM, **TGM2**, **MYH10**, RL30, RM39, **SEPT8**, ESTD
UniProt keywords	Phosphoprotein	35	TS101, ACDSB, **MYH9**, **1433B**, PSME3, AT2A3, TPMT, DNJC3, PPR1A, TWF2, RS26, PXDC2, **UCHL1**, **ARHL1**, **APOA1**, STT3B, DC1L1, **WEE2**, HTR5A, MAP2, **H14**, RCAN1, EEA1, THEM6, ODBA, RENT1, **6PGD**, NACAM, **MYH10**, ALPK3, RL30, **SEPT8**, MTM1, SRBS2, **FIBG**

*Note*: Proteins that were inversely associated with aerobic capacity are shown in bold.

Abbreviations: GO, gene ontology; PPI, protein–protein interactions.

### 3.3 Association of muscle proteome with aerobic capacity: wild‐type mice

**FIGURE 3:**
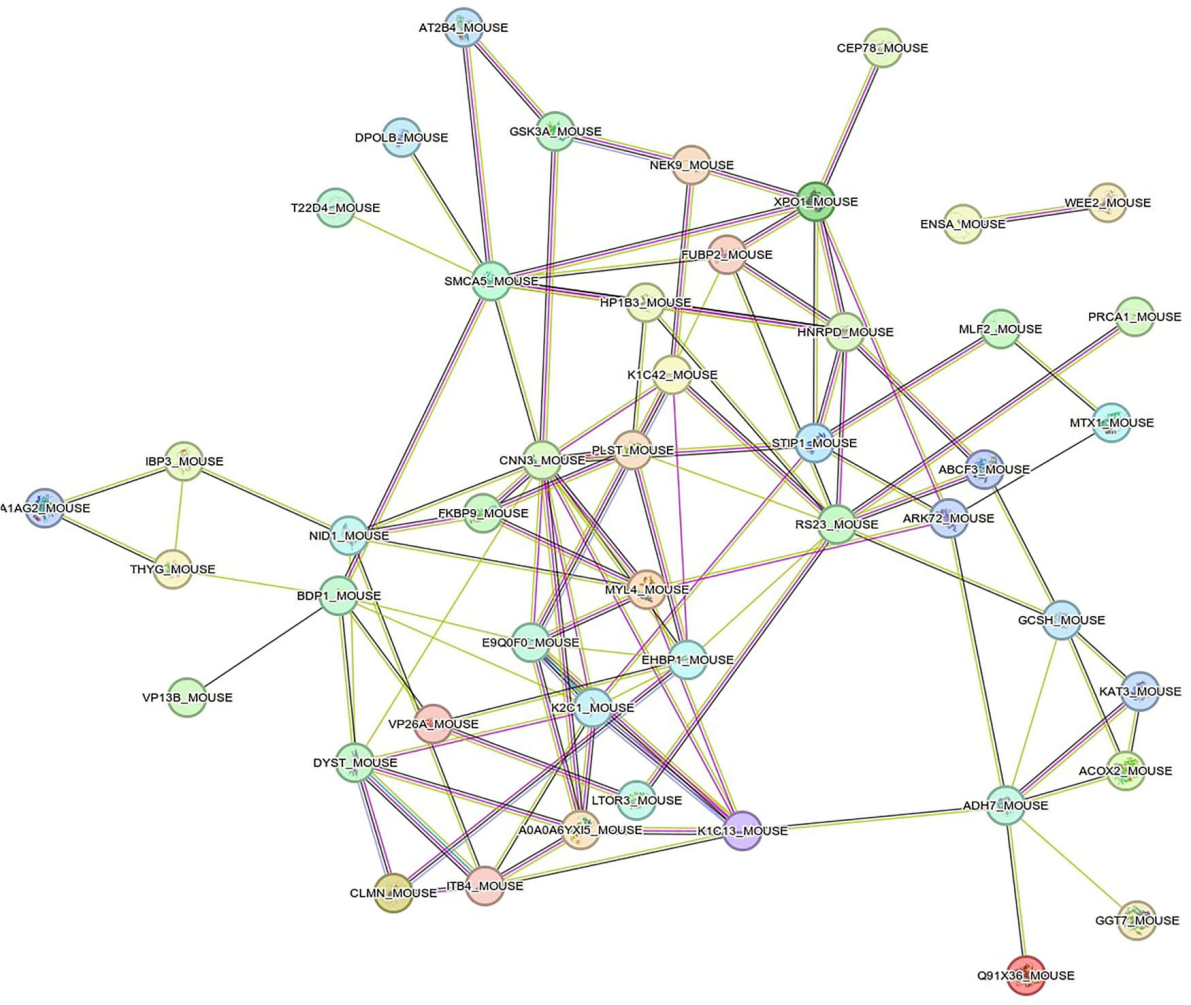
PPI network associated with aerobic capacity in wild‐type mice. Each node represents a protein and the edges colour the type of relationship between each protein (i.e., node in the network). Purple line, experimental evidence; yellow line, textmining evidence; light blue line, database evidence; black line, coexpression evidence. No meaning is given to the node colour, which was assigned by default in the STRING database. PPI, protein–protein interaction; STRING, Search Tool for the Retrieval of Interacting Genes/Proteins.

A total of 3144 proteins were detected in the skeletal muscle samples from wild‐type mice. AC was significantly associated with 61 proteins in skeletal muscle (33 positive (*r* ranging from 0.71 to 0.94) and 28 inverse (–0.71 to –0.90) associations, with individual *P*‐values ranging from 0.0004 to 0.049) (Figures 2b and 3 and Supplementary File 3). When further GO analysis was performed using the STRING database (http://string‐db.org/) (Szklarczyk et al., 2023) as well as manual curation of the literature, it was observed that these proteins are mainly involved in the following:

Cytoskeleton structure (principally, intermediate filaments): keratin, type I cytoskeletal 13 (K1C13), and 42 (K1C42); keratin, type II cytoskeletal 1 (K2C1); dystonin (DYST); keratin 78 (E9Q0F0); calmin (CLMN); plastin‐3 (PLST); calponin‐3 (CNN3); integrin β‐4 (ITGB4); myosin light chain 4 (MYL4); and EH domain binding protein 1 (EHBP1).

Cell cycle regulation: serine/threonine‐protein kinase Nek9 (NEK9); exportin‐1 (XPO1); homeobox‐containing protein 1 (HMBX1); transcription factor TFIIIB component B (BDP1); SWI/SNF‐related matrix‐associated actin‐dependent regulator of chromatin subfamily A member 5 (SMCA5); and Wee1‐like protein kinase 2 (WEE2).

Endocytic trafficking: vacuolar protein sorting‐associated protein 13B (VP13B); VPS26 retromer complex component A (VPS26A); EHBP1; and ragulator complex protein LAMTOR (LTOR3).

Figure 3 shows the PPI network (number of nodes = 52; number of edges = 105, expected number of edges = 78; PPI enrichment—*P*‐value = 0.002). These proteins were enriched in the GO biological process ‘intermediate filament cytoskeleton organization’, as well as in the GO cellular component terms ‘supramolecular complex and fibre’ (FDR = 0.046 and 0.045, respectively; Table 2). Proteins were identified with UniProt terms ‘phosphoprotein’ and ‘intermediate filament’ (FDR = 0.020 and 0.00075, respectively). The node degree ranged from 0 to 14 (mean = 4). Two proteins (calponin 3 (CNN3) and ribosomal protein 23 (RS23)) showed a node degree ≥10 (Supplementary File 3).

**TABLE 2:** Functional enrichment analyses of proteins associated with aerobic capacity in wild‐type mice using Search Tool for the Retrieval of Interacting Genes/Proteins (STRING) database (Szklarczyk et al., 2023).

Category	Selected terms	Number of proteins in each term	Matching proteins in the PPI network
GO process	Intermediate filament cytoskeleton organization	5	**K1C13**, **K1C42**, **K2C1**, **E9Q0F0**, DYST
GO component	Supramolecular complex	13	**K1C13**, FUBP2, **K1C42**, **K2C1**, **DPOLB**, GSK3A, **XPO1**, **MYL4**, EHBP1, **PLST**, **AT2B4**, **E9Q0F0**, DYST
GO component	Supramolecular fibre	11	**K1C13**, **K1C42**, **K2C1**, **DPOLB**, GSK3A, **MYL4**, EHBP1, **PLST**, **AT2B4**, **E9Q0F0**, DYST
UniProt keywords	Intermediate filament	5	**K1C13**, **K1C42**, **K2C1**, **E9Q0F0**, DYST
UniProt keywords	Phosphoprotein	32	**ABCF3**, FUBP2, T11L2, IBP3, **K2C1**, STIP1, GGT7, **FKBP9**, **N4BP1**, CEP78, **WEE2**, SMCA5, VP13B, **NEK9**, ENSA, GSK3A, ARK72, PRCA1, **VP26A**, **T22D4**, **XPO1**, EHBP1, CLMN, **PLST**, **AT2B4**, ACOX2, ITB4, HP1B3, **HNRPD**, **HMBX1**, **MLF2**, DYST

*Note*: Proteins that were inversely associated with aerobic capacity are shown in bold.

Abbreviations: GO, gene ontology; PPI, protein–protein interactions.

**FIGURE 4:**
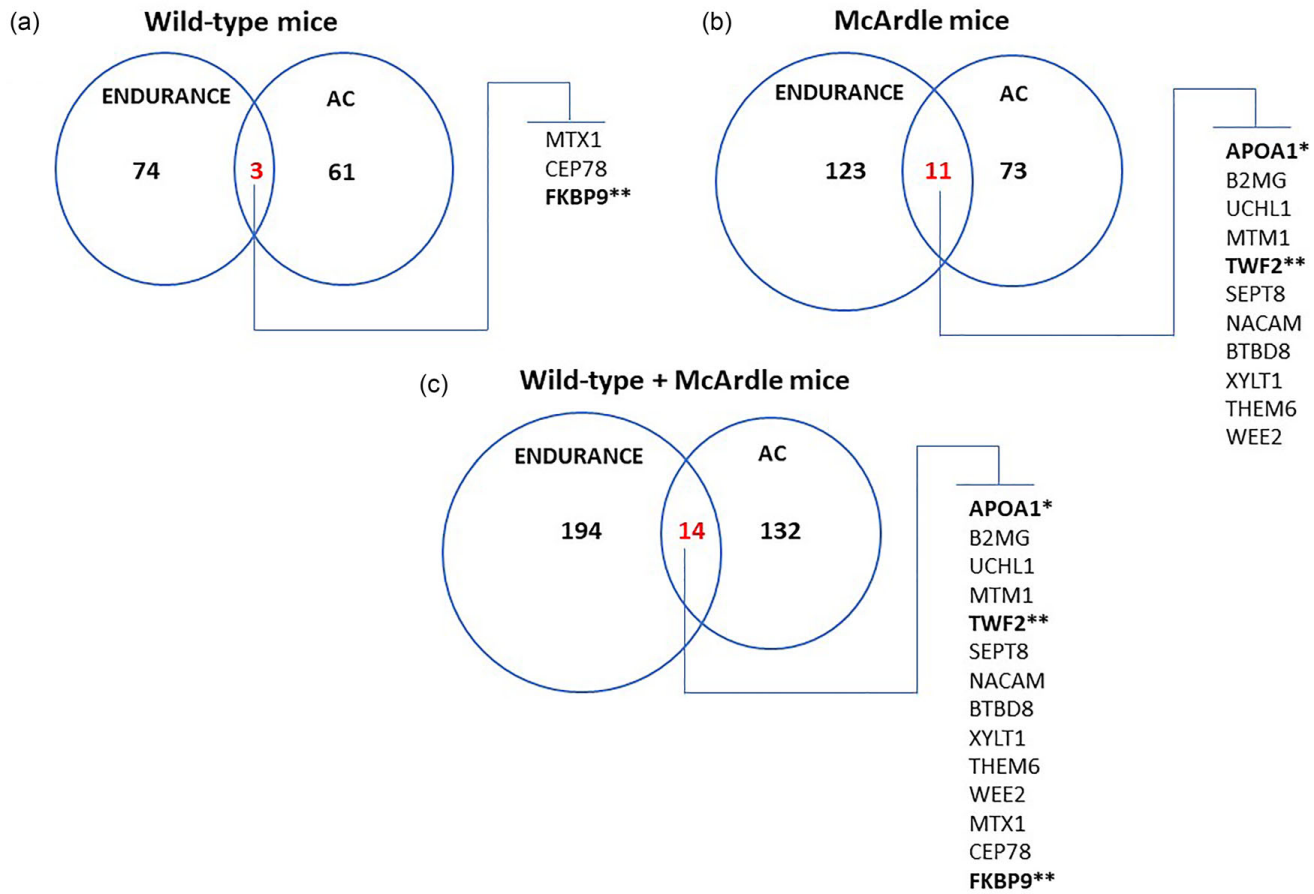
Venn diagrams showing the number of proteins in wild‐type (a) and McArdle (b) mice, or in both (c) that were (i) associated with AC and also (ii) previously found to be altered by aerobic training (Fiuza‐Luces et al., 2018)—the number of overlapped proteins is marked in red (see text for protein abbreviations). *Protein identified in the PHENOPEDIA database (Yu et al., 2010) for type I and II diabetes; **proteins identified in an extensive meta‐analysis of aerobic exercise training studies (Amar et al., 2021). AC, aerobic capacity.

At the individual level, only two proteins (WEE2 and thyroglobulin (THYG)) were significantly associated (negatively and positively, respectively) with AC in the two mouse models. Additionally, only 11 (APOA1, B2MG, UCHL1, MTM1, twinfilin‐2 (TWF2), septin‐8 (SEPT8), nascent polypeptide‐associated complex subunit α, muscle‐specific form (NACAM), BTB domain containing 8 (BTBD8), xylosyltransferase 1 (XYLT1), thioesterase superfamily member 6 (THEM6) and WEE2) of the 73 muscle proteins (15%) associated with AC in McArdle mice were previously found to be associated with aerobic training in these mice (Fiuza‐Luces et al., 2018), versus three (metaxin‐1 (MTX1), centrosomal protein 78 (CEP78), and FKBP prolyl isomerase 9 (FKBP9)) of 61 proteins (5%) in the wild‐type animals (Fiuza‐Luces et al., 2018) (Figure 4). Furthermore, none of the three proteins (LIM and calponin‐homology domains 1 (LIMCH1), poly (ADP‐ribose) polymerase 1 (PARP1), and tigger transposable element derived 4 (TIGD4)) that were previously shown to be upregulated in both McArdle and wild‐type with aerobic training (Fiuza‐Luces et al., 2018) were associated with AC in either mouse group.

### 3.4 In silico analyses

**FIGURE 5:**
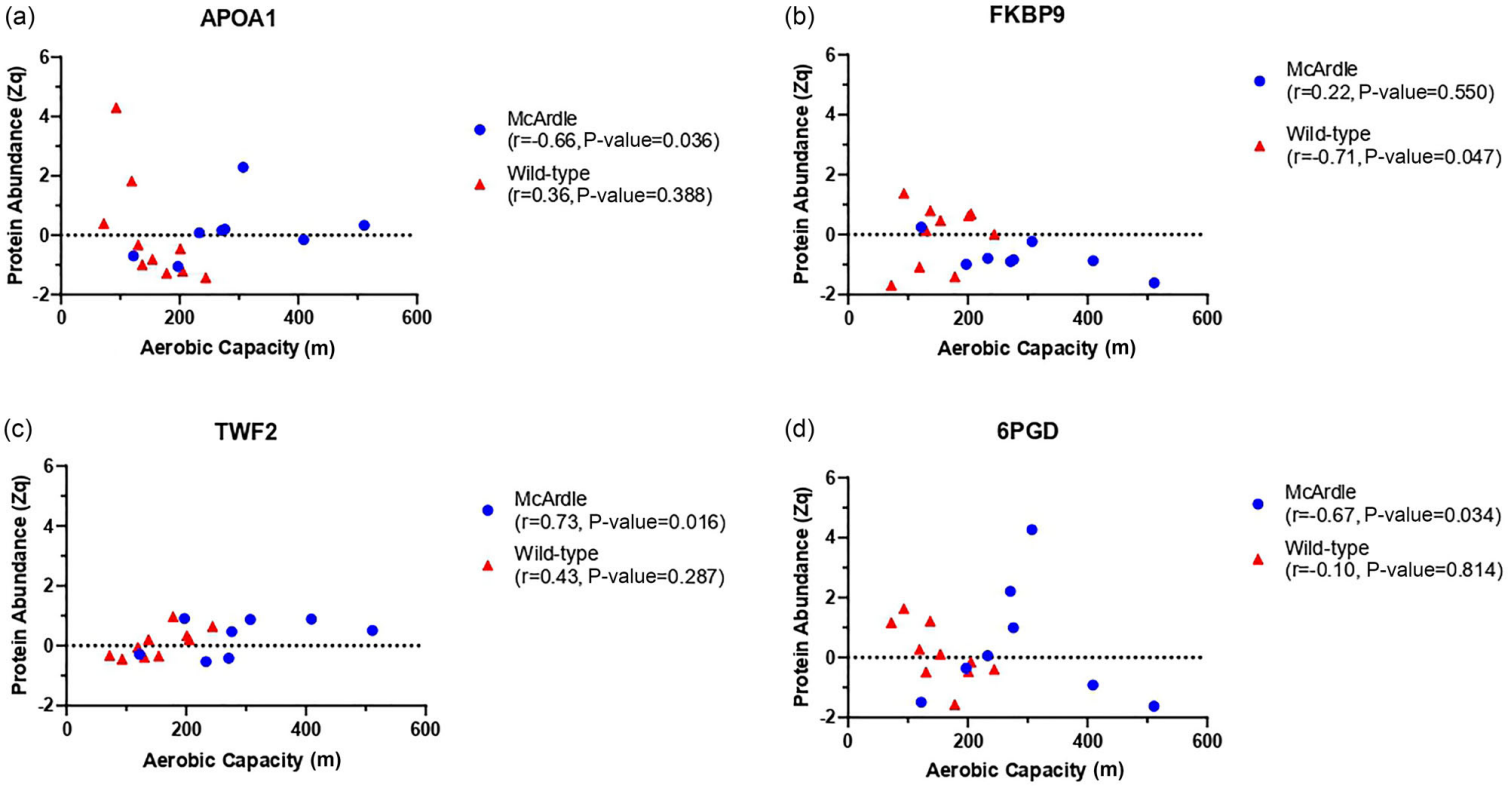
Plots of individual data points for three proteins associated with aerobic capacity and identified in our data mining approach (a, b, and c), as well as for one hub protein in the interaction network in McArdle mice (Figure 1) with relevant implications for glucose metabolism (d). Aerobic capacity is presented on the *x*‐axis, while protein abundance is indicated on the *y*‐axis.

The muscle proteins that were differentially expressed in patients with McArdle disease compared to healthy controls in our previous report with humans (García‐Consuegra et al., 2022) were not associated with AC in the skeletal muscle from the McArdle or wild‐type mice of the present study. Our in silico data mining approach identified: one protein, APOA1, significantly associated with type I and II diabetes in the PHENOPEDIA database; and two proteins, TWF2 and FKBP9, significantly associated with endurance training in https://www.extrameta.org/ (Amar et al., 2021) (Figure 5). Yet, for TWF2 and FKBP9, values of *I* ^2^ (i.e., the ratio of true heterogeneity to total observed variation, an indicator of statistical consistency; Higgins et al., 2003) in https://www.extrameta.org/ were higher than 70%, indicating that the *P*‐values obtained in this data resource may be inaccurate.

## 4 DISCUSSION

Using high‐throughout, high‐sensitivity proteome analyses at the muscle tissue level (i.e., >3000 proteins identified), our study provides preliminary evidence to support that the muscle proteome signature of AC differs with respect to the inherent energy transfer capacity of this tissue (i.e., abnormally low (McArdle) or ‘normal’ (wild‐type)). Indeed, at the individual level, only two proteins (WEE2 and thyroglobulin (THYG)) were significantly associated (negatively and positively, respectively) with AC in both mouse groups (vs. 73 and 61 different proteins separately associated with this variable in the McArdle and wild‐type group, respectively). Notably, we identified a higher number of proteins involved in lipid metabolism and mitochondrial function as well as in muscle regeneration and differentiation associated with AC in McArdle than in wild‐type animals. In the latter, the muscle proteome signature was more related to cytoskeleton reorganization and intermediate filaments. Concerning the finding of proteins involved in lipid metabolism for the McArdle group, we think this is an expected adaptation in an attempt to compensate, at least partly, for the inherent limitation in ATP generation from glycogen metabolism that characterizes this disease. In fact, we have previously shown that patients with this condition show an exceptionally high rate of maximal fat oxidation rate (MFO), which they attain at near‐maximal exercise capacity (Rodriguez‐Lopez et al., 2023). This unique fat oxidation capacity possibly reflects ‘an adaptation to compensate for the inherited blockade in glycogen metabolism’, with MFO rate being a potential limiting factor of exercise tolerance in this inherited disorder (Rodriguez‐Lopez et al., 2023). On the other hand, the fact that we found a higher number of proteins involved in mitochondrial function associated with AC in McArdle than in wild‐type animals would also reflect a biological adaptation—in this case, the excess glycogen depots cause mechanical disruption of the mitochondrial network inside the muscle fibres (as shown in affected mice and patients), which in turn could cause some degree of mitochondrial dysfunction, at least in those fibres with a higher capacity for glycogen accumulation (Villarreal‐Salazar et al., 2022).

Another important finding was that the AC‐related muscle proteome signature largely differs from that linked to previous aerobic training, in conditions of either low or normal AC. We believe that these findings might add potential insights into the molecular underpinnings of AC, since this parameter is dependent on the synergistic action of several tissues that participate in the oxygen transport cascade from the atmosphere to mitochondria, including not only the pulmonary and cardiovascular systems, but also the skeletal muscle tissue. Of note, however, the aerobic training programme that was previously performed by 50% of mice in each of the two groups was of low‐to‐moderate intensity, largely due to the fact that glycogen metabolism (which is needed to sustain intense exercise) is blocked in McArdle disease, and thus McArdle mice are unlikely to tolerate intense training loads—which, on the other hand, might raise the risk of rhabdomyolysis in these animals (Lucia et al., 2021). Further research is therefore needed to compare the muscle proteome signature associated with more intense training programmes.

Skeletal muscle cells convert chemical energy to physical work supporting locomotion and posture. Recently, a proteome study showed that circulating proteins related to skeletal muscle were associated with fibroblast activation protein (FAP) expression, which in turn was related to CRF levels in humans (Robbins et al., 2023). Also, FAP expression levels were upregulated with exercise training in murine skeletal muscle, and they were inversely associated with all‐cause mortality in three different human cohorts (Robbins et al., 2023). In the current study, we did not detect a significant association between muscle FAP and AC. However, we identified several proteins involved in skeletal muscle fibre differentiation that were associated, whether positively (RCAN1, SGCB, ALPK3) or negatively (MYH9, MYH10, ARHL1) with AC in McArdle—but not in wild‐type—mice. We therefore hypothesize that proteins related to muscle fibre differentiation and development may influence (whether positively or negatively) aerobic fitness in disease conditions that compromise energy transfer capacity at the skeletal muscle tissue level.

The ‘high’ number of proteins associated with AC that were identified in the ‘phosphoprotein’ term in both McArdle and wild‐type mice (35 and 32 proteins, respectively) are worth noting. For this effect, one study undergoing phosphoproteome analysis in skeletal muscle from rats, mice and humans showed a novel exercise‐induced mechanism through which AMP‐activated protein kinase (AMPK) regulates skeletal muscle calcium homeostasis (Nelson et al., 2019), which is impaired in the context of McArdle disease, as discussed further below. On the other hand, only two proteins were significantly associated with AC in both mouse groups, WEE2 and THYG (negative and positive association, respectively). This finding would suggest that these proteins might play a certain role in determining AC irrespective of individual peak capacity for energy transfer (i.e., whether limited or normal). More research is needed to unveil a potential role of WEE2 at the muscle level with respect to AC. This protein is predicted to enable the activity of protein tyrosine kinases, a group of enzymes that catalyse the transfer of the phosphate group of ATP to tyrosine residues in protein substrates and have been implicated in the regulation of a variety of biological responses such as cell proliferation, migration, differentiation and survival, and might also play important roles in the development of numerous disease conditions, including immunodeficiency, atherosclerosis, psoriasis, osteoporosis, diabetes and cancer (Blaukat, 2007). In turn, the potential role of THYG expression at the muscle tissue level is interesting and deserves further scrutiny, especially when considering that this hormone is specific to follicular cells of the thyroid gland, where it is secreted to the colloid and iodinated for the production of thyroid hormones. In any case, it is well known that thyroid function plays a major role in exercise tolerance including skeletal muscle function, with low levels linked to impaired OXPHOS in the mitochondria of this tissue (Ylli et al., 2020).

The association between AC and muscle levels of proteins involved in calcium handling is in line with the pathophysiology of the disease, which is characterized by an inherent impairment in this process, since high rates of glycogen‐derived ATP production are needed for adequate removal of calcium from the sarcoplasm back into the sarcoplasmic reticulum after each muscle contraction (Santalla et al., 2014). In fact, recent research has shown lower levels of sarcoplasmic/endoplasmic reticulum calcium ATPase 1 in the skeletal muscle tissue of patients with McArdle disease compared with controls (García‐Consuegra et al., 2022). On the other hand, the association of proteins involved in muscle fibre differentiation with AC in McArdle mice might reflect an adaptation to the state of muscle damage that characterizes this disease—even under resting conditions — and that affects predominantly IIA, IIX and IIA/IIX fibres versus the type I, I/IIA, IIX/IIB or IIB subtype (which are only mildly affected) (Krag, Pinós, Nielsen, Brull, et al., 2016). Muscle damage in McArdle disease is due to several mechanisms that contribute to structural muscle fibre fragility and membrane disruption, including downregulation of sodium–potassium pumps (which allow maintenance of cellular volume and integrity in muscle fibres), oxidative stress, or the mechanical stress imposed on the cytoskeleton by massive glycogen depots (Santalla et al., 2014; Villarreal‐Salazar et al., 2022). In this effect, some degree of re‐expression of non‐muscle (particularly, fetal) isoforms of glycogen phosphorylase might occur in the regenerating fibres of McArdle mice, especially in the aforementioned IIA, IIX and IIA/IIX fibres, allowing transient rescue of some residual enzyme activity (Gorin et al., 1989; Sato et al., 1977). This might facilitate, at least partly, glycogen‐derived energy to support regeneration and differentiation of muscle fibres in a context of continuous muscle damage.

Some hub muscle proteins (i.e., node degree ≥10) might be linked to the complex interplay between aerobic fitness and health/disease status such as 6PGD (Figures 1 and 5d), which was negatively associated with AC in McArdle mice. Interestingly, although 6PGD is primarily known for its involvement in the pentose phosphate pathway (where it forms ribulose 5‐phosphate from 6‐phosphogluconate), it also plays a role in the regulation of skeletal muscle glucose metabolism—increased 6PGD activity is a common intracellular adaptation that is present across disease states with an underlying pathology of insulin resistance and glucose intolerance (Lee‐Young et al., 2016). On the other hand, truncated APOA1 was negatively associated with AC in McArdle mice. Although APOA1 is the main apolipoprotein in plasma high‐density lipoproteins and has several well‐known cardioprotective functions, there is growing evidence for a pleiotropic role of this protein and its potential involvement in numerous non‐communicable diseases, such as cancer or neurological disorders (Cochran et al., 2021). In fact, the circulating levels of this truncated form of apolipoprotein A‐I transported by low‐density lipoproteins is increased in diabetes (Cubedo et al., 2015).

Our results should be interpreted as preliminary given the small sample size. In fact, the *P*‐values of correlation analyses did not reach statistical significance after correction for multiple hypothesis testing. Yet, we believe this limitation is justifiable when considering the remarkable difficulty in generating adult McArdle mice, owing to the high perinatal and postweaning mortality of these animals (Real‐Martinez et al., 2019). Additional limitations stem from the fact that we did not perform gas‐exchange measurements for direct ${\dot{V}}_{O_{2}\mathrm{peak}}$ assessment or record spontaneous physical activity levels. Furthermore, our proteome analyses were confined to the quadriceps muscle, which has a highly glycolytic phenotype. Future research should determine whether comparable findings are obtained in more oxidative muscles like the soleus or even in other glycolytic muscles. In this regard, although there are differences between muscles—even between those with a similar metabolic phenotype and with only subtle differences in proportion of fibre types, such as the quadriceps and tibialis anterior—overall both fast‐ and slow‐twitch muscles are affected by structural degeneration as well as by energy deficiency in McArdle mice (Krag, Pinós, Nielsen, Duran, et al., 2016). Along this line, previous research has indicated that the quadriceps muscle of McArdle mice shows more compensatory adaptations to counterbalance energetic deficiency (i.e., in terms of expression of proteins involved in glucose uptake, glycogen synthesis and glycolysis) than other muscles, whether these are predominantly glycolytic (tibialis anterior, extensor digitorum longus) or oxidative (soleus) (Krag, Pinós, Nielsen, Duran, et al., 2016). On the other hand, identification of the proteome of other skeletal muscles, notably the diaphragm, would have provided broader insights, for instance regarding ventilatory muscle efficiency. Finally, future studies should perform gene expression and proteome analyses at the single‐cell level in models of inherently low AC (such as McArdle disease) to gain a better understanding of the molecular mechanisms behind the interplay between aerobic fitness and health (or resistance to disease).

### 4.1 Conclusion

Our findings suggest that in McArdle mice the ‘muscle proteome signature’ associated with AC is mostly related to muscle fibre differentiation/development, lipid oxidation, mitochondrial function and calcium handling, with other biological processes (such as cytoskeleton structure) involved in wild‐type mice. Of note, this proteome signature seems to largely differ from that linked to an aerobic training background. Although more research is needed (notably with more intense training programmes), our findings might shed light on the possible molecular mechanisms underlying aerobic fitness in healthy and pathological conditions.

## AUTHOR CONTRIBUTIONS

Conceptualization: Alejandro Lucia and Abel Plaza‐Florido. Formal analysis: Abel Plaza‐Florido, Alejandro Santos‐Lozano, Susana López‐Ortiz, and Carmen Fiuza‐Luces. Funding acquisition: Alejandro Lucia. Investigation (experiments): Tomàs Pinós and Carmen Fiuza‐Luces. Methodology: Tomàs Pinós and Carmen Fiuza‐Luces. Project administration: Alejandro Lucia. Resources: Alejandro Lucia and Carmen Fiuza‐Luces. Supervision: Alejandro Lucia and Carmen Fiuza‐Luces. Writing—original draft: Abel Plaza‐Florido and Alejandro Lucia. Writing—review and editing: All authors. All authors have read and approved the final version of this manuscript and agree to be accountable for all aspects of the work in ensuring that questions related to the accuracy or integrity of any part of the work are appropriately investigated and resolved. All persons designated as authors qualify for authorship, and all those who qualify for authorship are listed.

## CONFLICT OF INTEREST

None declared.

## Supporting information



Supplementary File 1. Proteome database.

Supplementary File 2. Summary of proteomics results in McArdle mice.

Supplementary File 3. Summary of proteomics results in wild‐type mice.

## Data Availability

The data that support the findings of this study are publicly available.
